# A reconfigurable MTM-EMBG MIMO antenna array with solar panel integration for sustainable 5G networks

**DOI:** 10.1038/s41598-026-48125-x

**Published:** 2026-05-11

**Authors:** Saif Al-Attar, Mohammad Alibakhshikenari, Yazeed Mohammad Qasaymeh, Mohammed Abdulrahman Dawood Al-Obaidi, Bal S. Virdee, H. Benetatos, Nur Syazreen Ahmad, Astrit Krasniqi, Taha A. Elwi, Muhammad Akmal Chaudhary, Nisar Ahmad Abbasi, Patrizia Livreri, Takfarinas Saber

**Affiliations:** 1 School of Electrical and Electronic Engineering, Universiti Sains Malaysia, Nibong Tebal, 14300 Penang Malaysia; 2 LERO, The Research Ireland Centre for Software, College of Science and Engineering, School of Computer Science, University of Galway, Galway, H91 TK33 Ireland; 3 Department of Electrical and Electronics Engineering, Dogus University, Umraniye, Istanbul, 34775 Türkiye; 4 Department of Electrical and Electronics Engineering, College of Engineering, Majmaah University, Majmaah, 11952 Saudi Arabia; 5 Altinbaṣ University, Esentepe, Şişli Büyükdere Cd. No: 147, Beşiktaş/İstanbul, 34349 Türkiye; 6 Center for Communications Technology, London Metropolitan University, London, N7 8DB UK; 7 Department of Automation and Artificial Intelligence Engineering, College of Information Engineering, Al-Nahrain University, Baghdad, Iraq; 8 Department of Electrical and Computer Engineering, Ajman University, Ajman, UAE; 9 Department of Electrical Engineering, School of Systems Engineering, Bahrain Polytechnic, Isa Town, Bahrain; 10 Department of Engineering, University of Palermo, Palermo, 90128 IT Italy

**Keywords:** Metamaterial (MTM) antenna, Electromagnetic band-gap (EMBG) structures, Reconfigurable MIMO antenna, Solar-panel-integrated antennas, Composite right/left-handed (CRLH) structures, Sub-6 GHz 5G communications, Mutual coupling reduction, Energy-efficient antenna systems, Energy science and technology, Engineering, Optics and photonics

## Abstract

This paper presents a compact, reconfigurable sub-6 GHz MIMO antenna array integrated with a solar panel, targeting energy-efficient and sustainable 5G communication networks. The proposed design addresses the critical challenges of antenna performance degradation and mutual coupling that typically arise when antennas are integrated with photovoltaic structures. To overcome these limitations, the antenna employs a metamaterial (MTM) radiating patch composed of a 5 × 3 Hilbert-curve split-ring resonator (SRR) array, which enhances impedance bandwidth and gain through plasmonic resonance behavior. Additionally, a defected electromagnetic band-gap (EMBG) ground plane is introduced to suppress surface waves and back radiation, thereby improving radiation efficiency and isolation. A novel composite right/left-handed (CRLH) isolation wall is incorporated between antenna elements to achieve strong mutual coupling reduction within an ultra-compact footprint. Frequency reconfigurability is realized using PIN diodes, enabling dynamic control of the operating bands and radiation characteristics. The proposed two-element MIMO configuration is mounted beneath a solar panel, demonstrating negligible impact on photovoltaic I–V characteristics, while simultaneously providing a gain enhancement due to constructive electromagnetic interaction. The antenna operates over a wide frequency range between 2.7 and 6.0 GHz and beyond, with resonances around 3 and 5 GHz, achieving a maximum gain of approximately 7.3 dBi.

## Introduction

The rapid proliferation of fifth generation communication networks has significantly increased the energy consumption of wireless infrastructure, particularly at base stations, leading to higher operational costs and growing environmental concerns^1–4^. As 5G systems continue to expand, there is a strong need for solutions that improve spectral efficiency and antenna performance while reducing reliance on conventional grid power^5–8^. Traditional base stations consume considerable energy, contributing to increased operational expenses and carbon emissions^9^.

One promising approach to address these challenges is the integration of renewable energy sources, particularly solar panels, into wireless communication systems^10–13^. Solar powered base stations can reduce reliance on grid power, lower carbon emissions, and improve network resilience during power outages. However, integrating antennas with solar panels introduces several challenges, including electromagnetic interference, radiation efficiency degradation, impedance mismatch, and strong mutual coupling between antenna elements^14–16^. These challenges are especially critical in Multiple Input Multiple Output systems, where high isolation, wide bandwidth, and stable radiation characteristics are required for reliable 5G operation.

MIMO technology enables simultaneous transmission across multiple spatial channels, increasing data throughput and network capacity^15^. However, compact MIMO antenna systems often experience strong mutual coupling, reduced gain, and limited bandwidth, particularly in space constrained environments or when integrated with structures such as solar panels^16^. Various approaches have been proposed to mitigate these issues, including antenna orientation diversity, defected ground planes, and decoupling structures^17–21^. Despite these efforts, achieving compact size, wide bandwidth, high isolation, and energy efficient operation within a single antenna platform remains challenging.

Metamaterial based antenna designs have recently demonstrated strong potential for improving antenna performance^22–30^. Metamaterials enable engineered electromagnetic properties that support antenna miniaturization, bandwidth enhancement, surface wave suppression, and gain improvement. Split ring resonators and fractal geometries have been widely used to enhance antenna performance for wearable, portable, and sub six gigahertz wireless applications^28–30^. Similarly, electromagnetic band gap structures have been employed to suppress surface waves and reduce mutual coupling in MIMO antennas^26–29^.

Energy efficiency has also become an important design objective for next generation wireless networks^31–33^. Integrating solar panels with communication antennas provides a promising approach for sustainable 5G infrastructure. However, most reported solar integrated antenna systems focus primarily on energy harvesting, with limited attention to antenna reconfigurability, isolation enhancement, and compact MIMO implementation^32,33^. In addition, performance degradation caused by the presence of solar panels remains a concern, particularly for sub six gigahertz MIMO systems.

To address these challenges, this paper proposes a compact and reconfigurable solar panel integrated MIMO antenna array for sub six gigahertz 5G communication networks. The proposed design combines a Hilbert curve based metamaterial split ring resonator radiating patch, a defected electromagnetic band gap ground plane, and composite right left handed isolation walls to enhance bandwidth, increase gain, and suppress mutual coupling. PIN diodes are incorporated to enable frequency reconfigurability and dynamic control of the operating bands. The antenna array is mounted beneath a solar panel, and the electromagnetic interaction with the photovoltaic structure is experimentally investigated, demonstrating negligible impact on solar panel performance while maintaining stable antenna characteristics.

The proposed antenna operates from 2.7 GHz to 6 GHz and beyond, covering key sub six gigahertz 5G bands while achieving isolation better than − 15 dB and low envelope correlation suitable for MIMO operation. The design is validated through both simulation and measurement, confirming the effectiveness of the proposed metamaterial, EMBG, and CRLH integration strategy for sustainable and high performance 5G communication systems.

The remainder of this paper is organized as follows. Section II describes the antenna geometry and design details. Section III presents the characterization of the proposed CRLH structure. Section IV discusses the design methodology and parametric analysis. Section V examines the PIN diode switching scenarios. Section VI presents fabrication and experimental results. Section VII evaluates wireless channel performance. Section VIII provides a comparison with existing works, and Section IX concludes the paper.

### Antenna design and geometrical details

**Fig. 1:**
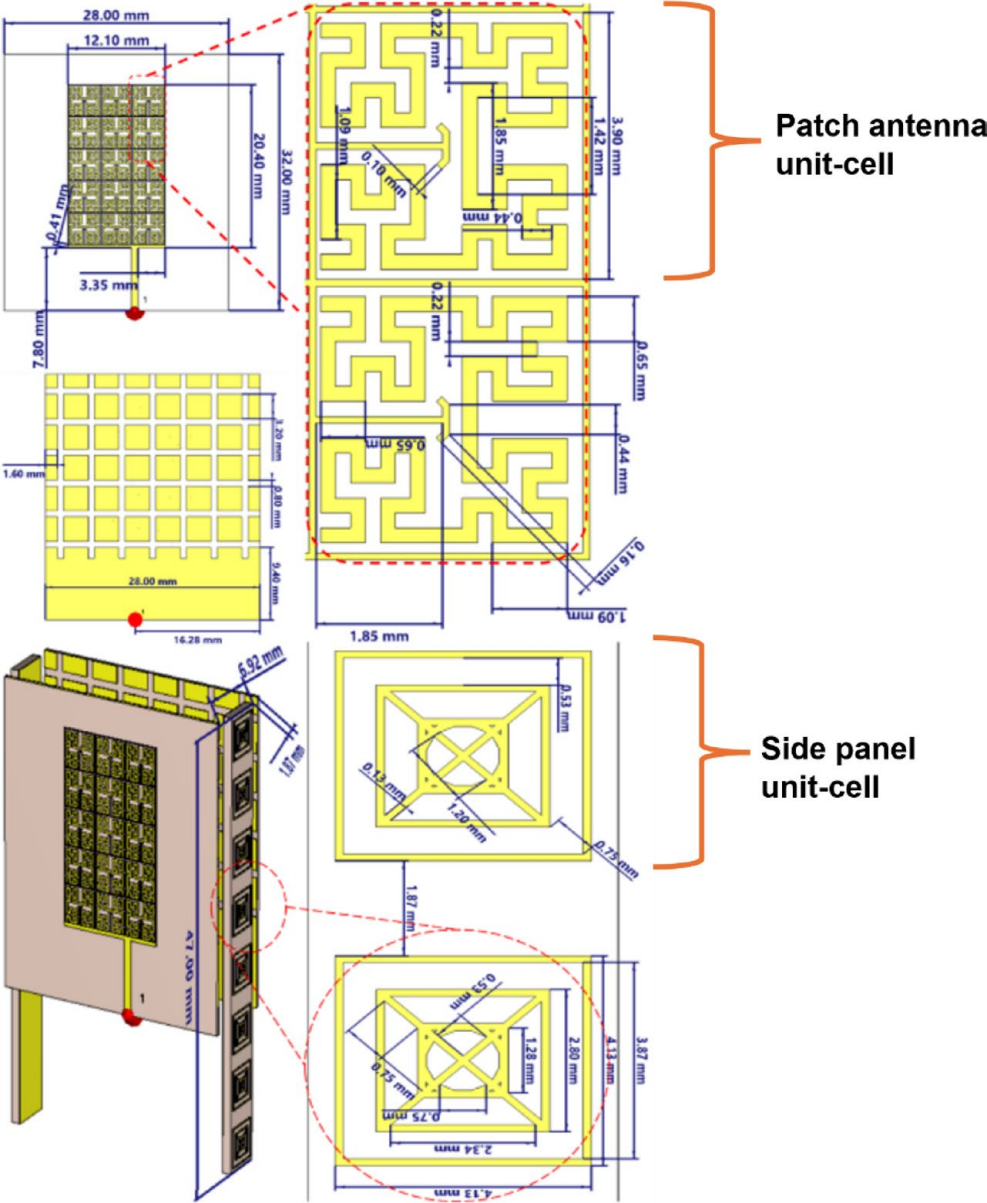
Geometrical details of the proposed antenna system that includes overall dimensions of the single antenna element with the 3 × 5 metamaterial unit cell array, enlarged view of the Hilbert curve split ring resonator based metamaterial unit cell with embedded T-shaped resonator, defected electromagnetic band gap ground plane configuration, three dimensional view of the proposed antenna element mounted beneath the solar panel, and geometrical details of the composite right left handed isolation base used between MIMO antenna elements.

The proposed antenna is designed based on a metamaterial radiating patch combined with a defected electromagnetic band gap ground plane in order to achieve wide bandwidth, enhanced gain, and reduced mutual coupling for compact MIMO applications. The geometrical configuration of the antenna is illustrated in Fig. 1, where the right side shows the single antenna element and the left side shows the MIMO array arrangement.

The radiating element is constructed from a metamaterial array etched on the microstrip patch, enabling plasmonic zero resonance behavior^11^. The MTM patch consists of a 3 × 5 array of unit cells, resulting in an overall compact size of 20.4 × 12.1 mm², as shown in Fig. 1(a). Each unit cell is formed by embedding a T shaped resonator inside a Hilbert curve split ring resonator structure.

Embedding the T-shaped resonator within the SRR introduces strong capacitive coupling, which contributes to bandwidth enhancement and surface wave suppression^12^, while maintaining improved radiation efficiency through better electromagnetic aperture matching with free space impedance^15^. A closed conductive loop surrounds the resulting structure to ensure effective surface current excitation from the radio frequency source. The radiating patch is printed on the top side of the dielectric substrate.

The bottom side of the substrate is partially metallized to form the ground plane and is defected using an electromagnetic band gap array, as depicted in Fig. 1. The proposed EMBG structure consists of a 5 × 7 unit cell matrix, designed to reduce back radiation and suppress surface wave diffraction from the substrate edges. Such suppression is particularly important for applications where radiation toward nearby objects or the human body must be minimized^16^.

The EMBG unit cells employ cross slot geometries that introduce composite right left handed characteristics, enabling effective suppression of skew waves on the back panel of the antenna^17^. As a result, backward radiation lobes are significantly reduced, leading to improved radiation efficiency and front to back ratio^18^.

To provide frequency agility and adaptive performance, two PIN diodes are integrated into the antenna structure. The diodes are positioned within the metamaterial patch to modify the effective current paths and resonant behavior of the antenna. By switching the PIN diodes between different ON and OFF states, the antenna operating bands can be dynamically controlled. The detailed effects of diode switching on impedance matching and gain performance are discussed in Section V.

For MIMO operation, two identical antenna elements are arranged in a compact configuration, as shown in Fig. 1. The elements are separated by a distance of 8.8 mm, and the spacing region is filled with rectangular composite right left handed isolation bases. These bases are introduced to suppress near field coupling and reduce mutual coupling between antenna elements.

The proposed CRLH bases have a thickness of 1 mm and are optimized to achieve isolation levels better than − 15 dB across the operating frequency band. This approach enables compact MIMO implementation while preserving radiation efficiency and bandwidth.

The proposed MIMO antenna array is mounted beneath a planar solar panel for self powered wireless communication applications. This placement is selected to avoid electromagnetic interference between the antenna and the photovoltaic structure. Since the operating direction of the solar panel differs from the radiation direction of the antenna array, mutual interference is minimized^18^. Experimental results presented in later sections confirm that the proposed antenna has negligible impact on the electrical characteristics of the solar panel while maintaining stable antenna performance.

### CRLH surface characterizations

The proposed composite right left handed surface is designed to operate as an isolation and surface wave suppression structure for the compact MIMO antenna configuration. The CRLH layer is implemented as a periodic unit cell structure printed on an FR4 substrate with a thickness of 0.8 mm. Each unit cell consists of concentric square rings combined with cross shaped conductive lines, forming a geometry capable of supporting both right handed and left handed electromagnetic propagation. The electromagnetic characteristics of the proposed structure are investigated using equivalent circuit modeling, full wave simulations, and effective medium parameter extraction.

**Fig. 2:**
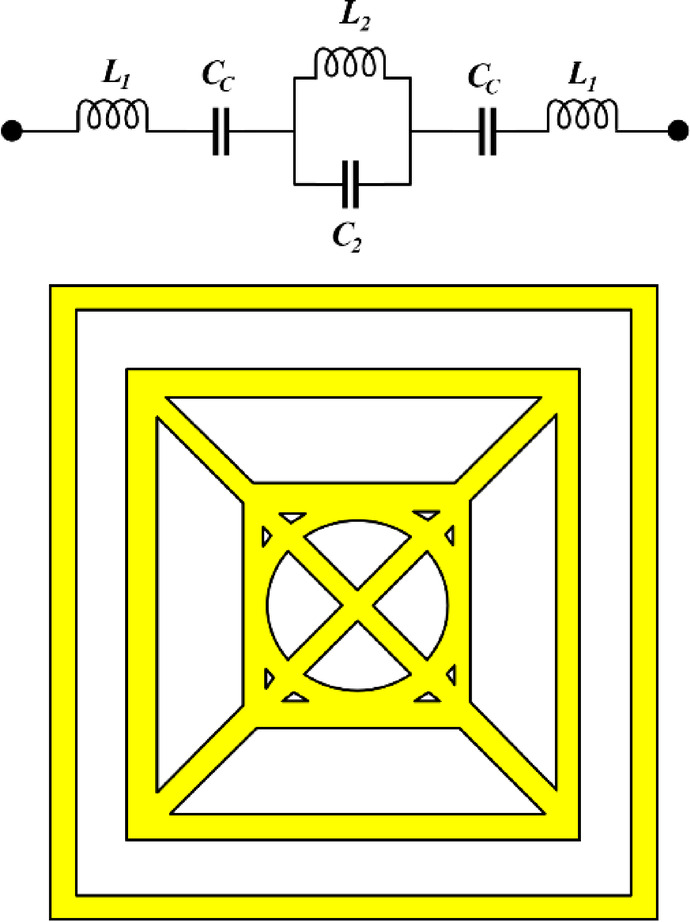
Circuit model of the proposed CRLH unit cell.

The geometry of the proposed CRLH unit cell and its corresponding equivalent circuit model are shown in Fig. 2. The circuit model is derived from the split ring resonator configuration reported in^19^, with a structural modification applied to the split ring section to introduce additional resonant behavior. The equivalent circuit consists of lumped inductive and capacitive elements that collectively describe the impedance and resonance characteristics of the unit cell.

In the proposed model, the conventional split ring resonator behavior is represented by a parallel *LC* branch composed of inductance *L*_*2*_ and *C*_*2*_. This branch defines the total impedance *Z*_T,2_ and the corresponding resonant frequency *f*_r,2_, which are expressed as

1 $$\:{Z}_{T,2}=\frac{1}{j\omega\:{C}_{2}}+j\omega\:{L}_{2}$$

2 $$\:{f}_{r,2}=\frac{1}{2\pi\:\sqrt{{L}_{2}{C}_{2}}}$$

To realize composite right left handed behavior and extend the operational bandwidth, two series *LC* branches composed of inductance $\:{L}_{1}\:$and capacitance $\:{C}_{c}\:$are introduced. These series branches define an additional total impedance $\:{Z}_{T,1}\:$and a band pass resonant frequency $\:{f}_{r,1}$, given by

3 $$\:{Z}_{T,2}=j\omega\:{L}_{1}+\frac{1}{j\omega\:{C}_{c}}$$

4 $$\:{f}_{r,2}=\frac{1}{2\pi\:\sqrt{{L}_{1}{C}_{c}}}$$

By combining the parallel and series resonant branches, the overall impedance response of the unit cell can be expressed as

5 $$\:{Z}_{T}={Z}_{T,1}\:\parallel\:\:\:{Z}_{T,2}$$

This combined impedance response enables the coexistence of right handed and left handed propagation modes, which is a defining feature of CRLH structures.

The extracted lumped element values used in the equivalent circuit model are summarized in Table 1, where the inductance and capacitance values are obtained through numerical optimization to match the full wave simulation results.

**Table 1:** Circuit model of the proposed unit cell.

Parameters	Value
*L*_*1*_	12.2 nH
*C*_*c*_	5 pF
*L*_*2*_	0.1 nH
*C*_*2*_	2.7 pF
*C*_*c*_	1.1 pF

**Fig. 3:**
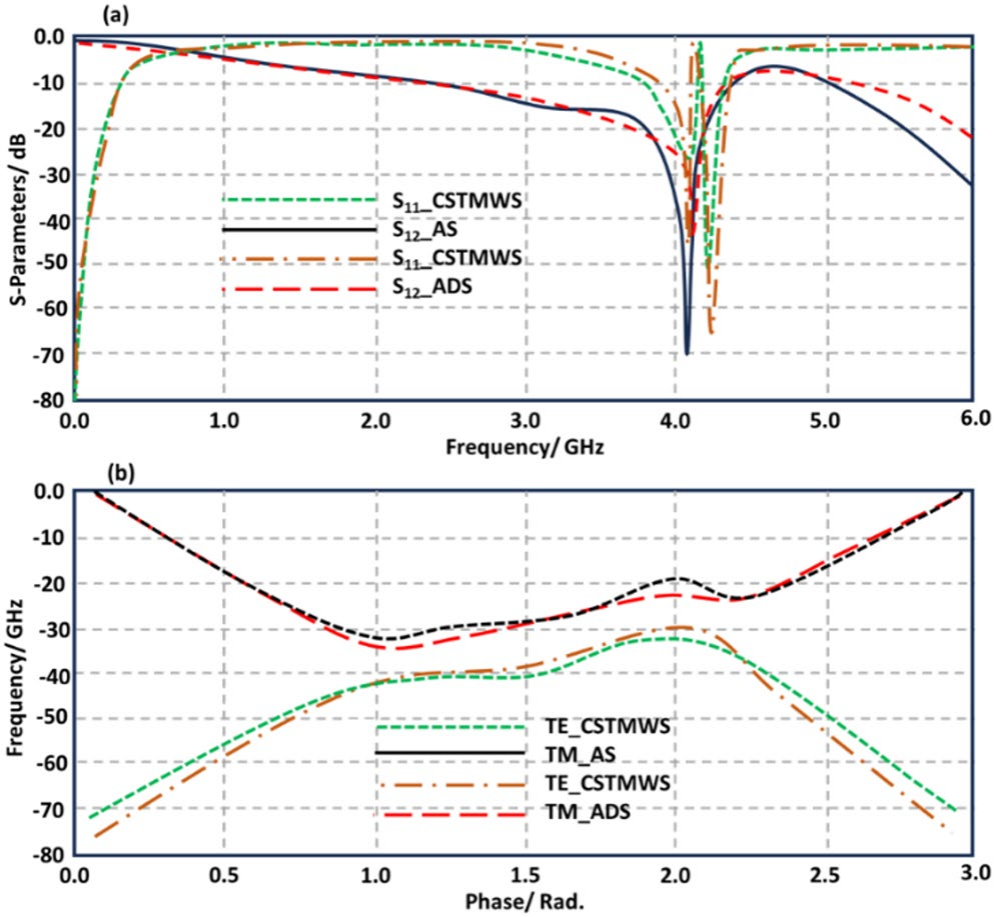
Electromagnetic characterization of the proposed MTM unit cell: (**a**) comparison between the circuit model and CST simulated S-parameters, showing strong agreement and a primary resonance near 4.1 GHz; and (**b**) dispersion diagram extracted using the eigenmode solver, confirming the MTM behavior across the first Brillouin zone.

The electromagnetic behavior of the proposed CRLH unit cell is evaluated in terms of scattering parameters and dispersion characteristics. The S parameters obtained from full wave simulations using CST Microwave Studio are compared with those calculated using the equivalent circuit model implemented in ADS, as shown in Fig. 3(a). A strong agreement between the two approaches is observed, confirming the validity of the proposed circuit representation.

As illustrated in Fig. 3(a), the unit cell exhibits a pronounced transmission null around 4 GHz, where the transmission coefficient magnitude drops below minus 35 dB, indicating strong attenuation and effective suppression of surface wave propagation. The stop band extends approximately from 3 GHz to 8 GHz when considering a minus 10 dB transmission threshold, demonstrating wideband isolation capability.

The dispersion characteristics of the unit cell are shown in Fig. 3(b), where frequency is plotted as a function of phase within the first Brillouin zone. The dispersion diagram is extracted using the eigenmode solver in CST Microwave Studio and is compared with analytical results derived from the equivalent circuit model. Both transverse electric and transverse magnetic modes are presented. The close agreement between numerical and analytical results confirms the presence of left handed and right handed propagation regions, validating the CRLH nature of the proposed structure.

**Fig. 4:**
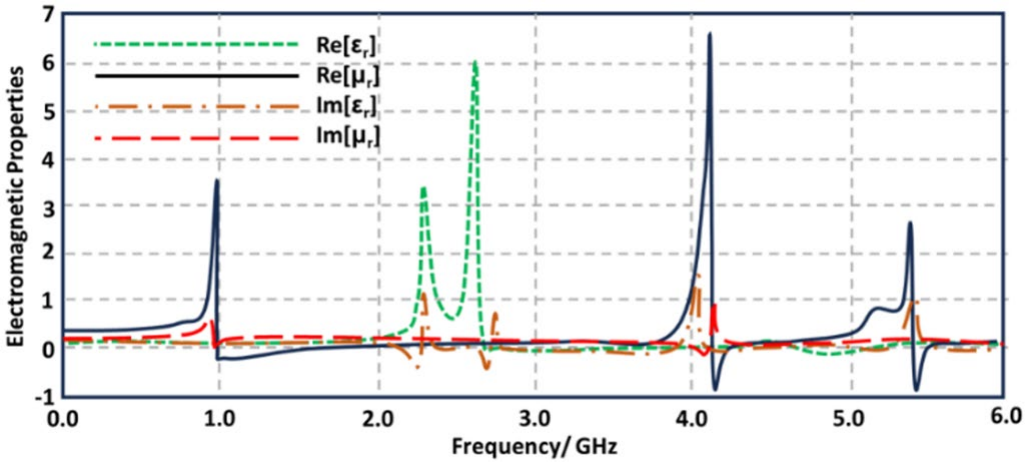
Extracted effective permittivity (*ε*_*r*_) and permeability (*µ*_*r*_) of the proposed MTM unit cell using the NRW method, illustrating multiple single-negative regions across the operating frequency range. These regions support surface wave suppression and improved radiation performance for wearable applications.

To further characterize the electromagnetic behavior of the proposed CRLH unit cell, the effective permittivity and permeability are extracted using the Nicolson Ross Weir method described in^23^. The extracted parameters are shown in Fig. 4.

The results indicate that the proposed unit cell exhibits multiple single negative frequency regions. Specifically, negative permittivity or permeability is observed approximately from 1.6 to 3 GHz and from 4.5 to 6 GHz, within the frequency region of interest. These single negative bands contribute to strong suppression of surface waves and back radiation, which is essential for improving antenna isolation and radiation efficiency.

The existence of these frequency selective single negative regions confirms the effectiveness of the proposed CRLH surface as an isolation base for compact MIMO antenna systems. These characteristics directly support the observed reduction in mutual coupling and enhancement in forward radiation performance discussed in subsequent sections.

### Design methodology

The design methodology follows a progressive evolution process to demonstrate the effect of each structural modification on antenna performance. The design begins with a conventional patch configuration and gradually incorporates metamaterial inclusions and electromagnetic band gap structures to achieve wide bandwidth, multiband operation, and improved impedance matching.

#### Reference patch without SRR and EMBG

**Fig. 5:**
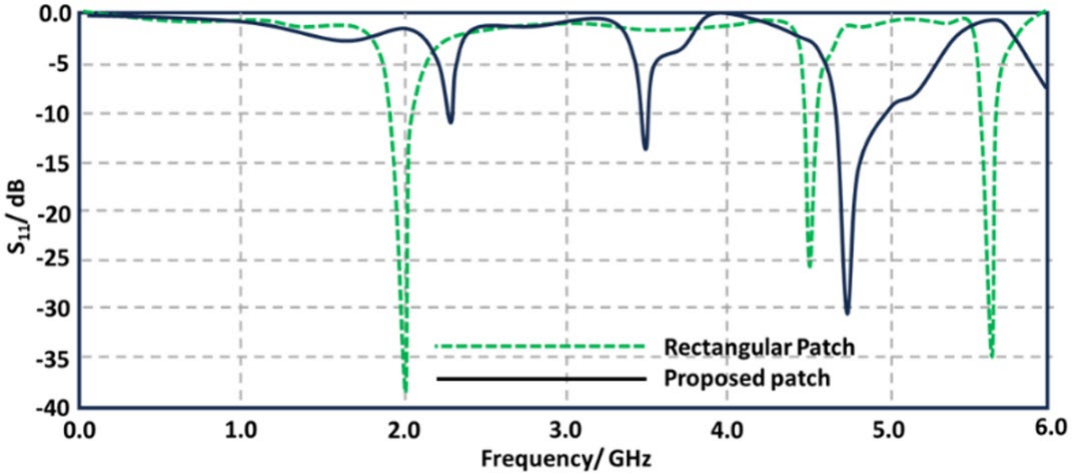
Reflection coefficient comparison between a conventional rectangular patch antenna and the proposed mesh based patch without SRR inclusions and without EMBG ground plane.

The initial stage considers a rectangular microstrip patch with a mesh based geometry formed by etching a square region from the metallic patch layer, as shown in Fig. 5. In this configuration, neither the split ring resonator inclusions nor the EMBG ground plane are introduced. This reference structure is used to evaluate the fundamental impedance behavior of the antenna.

The simulated reflection coefficient responses of the conventional rectangular patch and the proposed mesh based patch are compared in Fig. 5. The conventional patch exhibits narrowband behavior, while the mesh geometry produces improved impedance matching and additional resonance modes. This improvement results from the increased current path length and distributed capacitive loading introduced by the mesh structure.

#### Patch with SRR inclusions and without EMBG

To improve the impedance bandwidth and introduce additional resonant modes, Hilbert curve split ring resonator unit cells are embedded within the etched regions of the reference mesh patch, forming a metamaterial array.

**Fig. 6:**
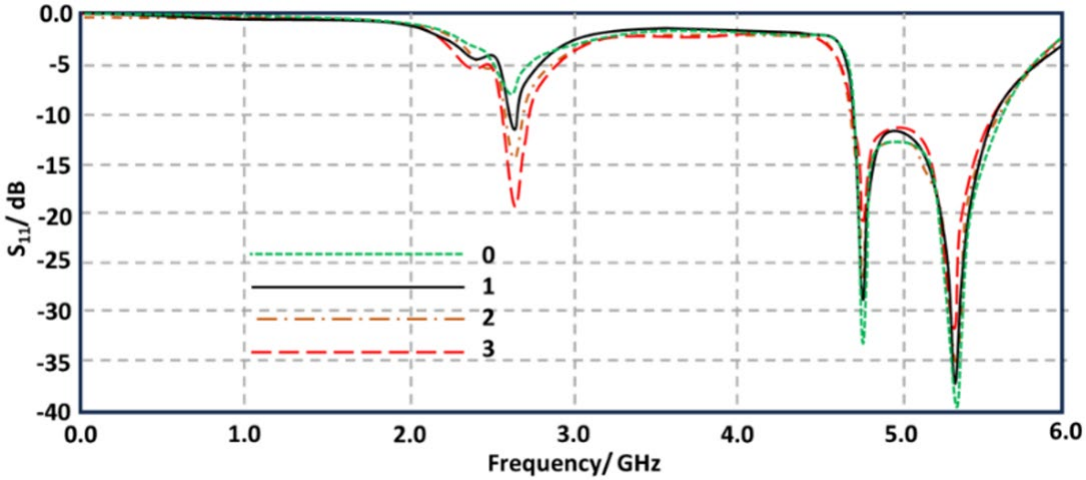
Reflection coefficient responses of the proposed antenna with embedded Hilbert curve split ring resonator inclusions for different fractal orders from zeroth to third order.

In the second stage, Hilbert curve split ring resonator unit cells are embedded within the etched regions of the patch to form a 3 × 5 metamaterial array, while the ground plane remains unchanged. The influence of these inclusions on the reflection coefficient is illustrated in Fig. 6.

Figure 6 shows the reflection coefficient responses for different fractal orders of the Hilbert curve SRR from zeroth to third order. Increasing the fractal order improves impedance bandwidth and introduces additional resonances. The antenna exhibits a resonance near 2.7 GHz and an extended operating region between approximately 4.7 GHz and 5.75 GHz. This behavior arises from the additional inductive and capacitive coupling created by the fractal SRR geometry, which produces multiple resonant current paths.

#### Patch with SRR inclusions and EMBG ground plane

**Fig. 7:**
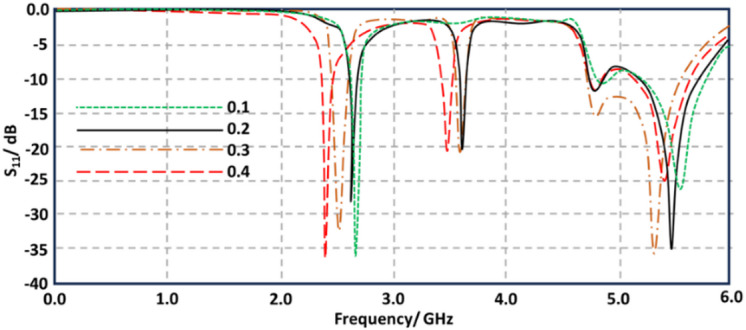
Effect of electromagnetic band gap ground plane unit cell separation distance on the reflection coefficient of the proposed antenna for separation values of 0.1 mm to 0.4 mm.

In the final stage, an EMBG structure is introduced on the ground plane to further improve antenna performance. The combined effect of the metamaterial radiating patch and the EMBG ground plane is presented in Fig. 7.

Figure 7 shows the reflection coefficient for different separation distances between adjacent EMBG unit cells, represented by parameter l, varied from 0.1 mm to 0.4 mm. The results indicate that the EMBG configuration significantly influences impedance matching and resonance characteristics. As the separation distance increases, the resonance frequencies shift and the depth of the reflection coefficient minima changes.

An optimal separation distance of 0.2 mm provides the best impedance matching and widest operating bandwidth, with strong resonances around 2.45 GHz and 3.5 GHz and an additional wideband response at higher frequencies. The improvement is mainly attributed to surface wave suppression and reduced back radiation introduced by the EMBG structure.

Overall, the design evolution demonstrates that the combined use of Hilbert curve metamaterial inclusions and the EMBG ground plane is essential for achieving wideband and multiband antenna performance.

### PIN diode switching scenarios

In this section, the effect of PIN diode switching on antenna impedance matching and gain is evaluated. Two PIN diodes are integrated into the radiating structure to modify the effective current paths and electrical length of the metamaterial patch, enabling frequency reconfiguration. With two diodes, four switching cases are possible, denoted as 00, 01, 10, and 11, where 0 and 1 represent the OFF and ON states of each diode, respectively.

#### Impedance matching performance

**Fig. 8:**
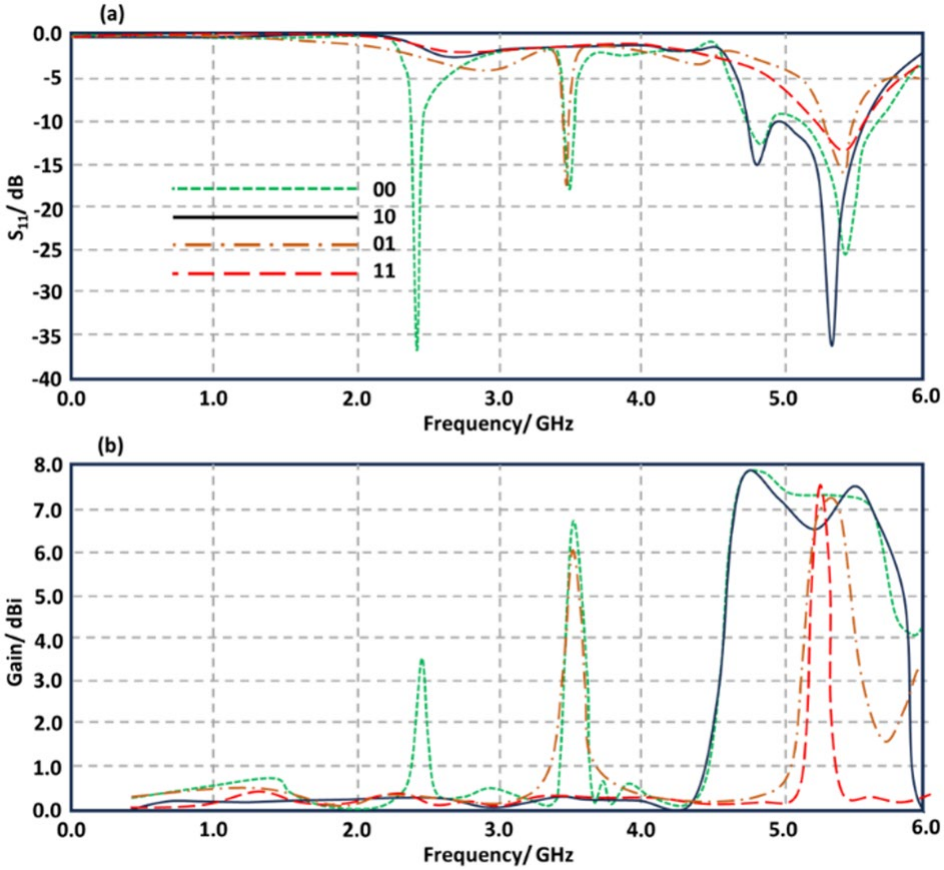
Effect of PIN diode switching on antenna performance: (**a**) reflection coefficient responses for the four switching states 00, 01, 10, and 11, and (**b**) corresponding antenna gain variations as a function of frequency.

The simulated reflection coefficient responses for the four switching scenarios are shown in Fig. 8(a). The results confirm that changing the diode states significantly alters the resonant modes and impedance matching depth.

For the 00 state, a strong resonance is observed at the lower band around 2.45 GHz, and additional resonant behavior appears in the upper band, including a wide response in the approximate range from 4.6 GHz to 5.4 GHz. When the switching state changes to 01 or 10, the resonance structure changes noticeably, where the lower band resonance is suppressed or weakened and the response becomes dominated by higher frequency resonances. In the 11 state, the impedance matching becomes more selective, concentrating the response mainly at the upper portion of the band with reduced multiband behavior.

#### Gain performance

The corresponding gain responses are presented in Fig. 8(b). In general, gain peaks occur at frequencies where impedance matching is strong. For the 00 state, the antenna shows multiple gain peaks, including a higher gain region in the upper band where the gain approaches about 7 to 8 dBi. For the 01 and 10 states, gain is significantly reduced at several frequencies due to suppression of resonant modes. This reduction is attributed to increased stored energy and additional losses associated with the diode loading and switching condition^25^. In the 11 state, gain becomes concentrated near the high frequency resonance, yielding a narrower usable band.

The slight gain reduction observed in some switching states is mainly attributed to the parasitic resistance and capacitance introduced by the PIN diodes, which increase ohmic loss and reactive loading in the radiating structure, representing a typical trade off between frequency reconfigurability and radiation efficiency in switch based antenna designs.

#### Quantitative summary of switching scenarios

To summarize the switching impact concisely, the resonance frequencies and corresponding gain values for each diode state are listed in Table 2.

The results in Fig. 8; Table 2 confirm that PIN diode switching provides effective control over the antenna resonant modes and gain response, enabling the proposed antenna to switch between multiband operation and more selective high band operation. The gain degradation observed in some switching states is consistent with additional loss and reactive energy storage introduced by diode loading^25^.

**Table 2:** Antenna performance for different PIN diode switching states.

Scenarios	Frequency resonance (GHz)	Gain (dBi)
00	2.45, 3.5, 4.6–5.4	3.5,5.2, 5.5
01	4.6–5.4	0.011, 0.001, 5
10	3.5, 5.4	5, 5.5
11	5.4	5.7

## Results and discussion

This section presents the experimental validation and performance analysis of the proposed antenna design, including single element measurements, MIMO array characterization, and the impact of solar panel integration. The simulated results obtained using CST Microwave Studio are compared with measured data to verify the accuracy and robustness of the proposed design.

### Single antenna fabrication and measurement

**Fig. 9:**
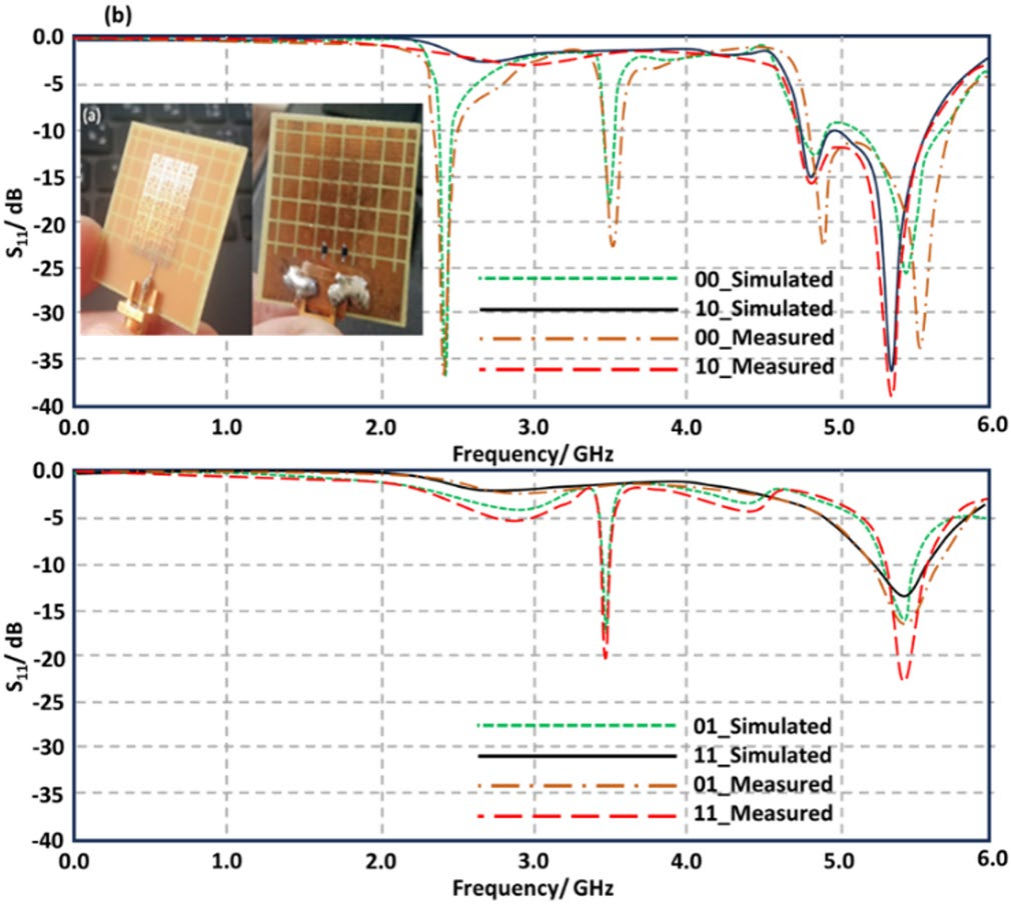
Single antenna experimental validation: (**a**) fabricated prototype with integrated PIN diodes, and (**b**) comparison between simulated and measured reflection coefficient responses for different diode switching states.

The fabricated prototype of the single antenna element is shown in Fig. 9(a). The antenna is realized using nano printing technology on an FR4 substrate that is treated inside a plasma reactor to reduce dielectric losses and improve surface adhesion. Two PIN diodes are soldered onto the radiating patch to enable frequency reconfiguration. The antenna is measured using a vector network analyzer (model 37347 A) to evaluate the reflection coefficient under different switching conditions.

The measured and simulated reflection coefficient responses are compared in Fig. 9(b) for representative switching states. A good agreement between simulation and measurement is observed across the entire frequency range. Minor discrepancies in resonance depth and frequency shift are attributed to fabrication tolerances, soldering effects, biasing wires, and the parasitic inductance of the PIN diode leads. Despite these practical nonidealities, the measured results confirm the validity of the simulated antenna behavior and demonstrate stable multi band operation.

### MIMO antenna performance

**Fig. 10:**
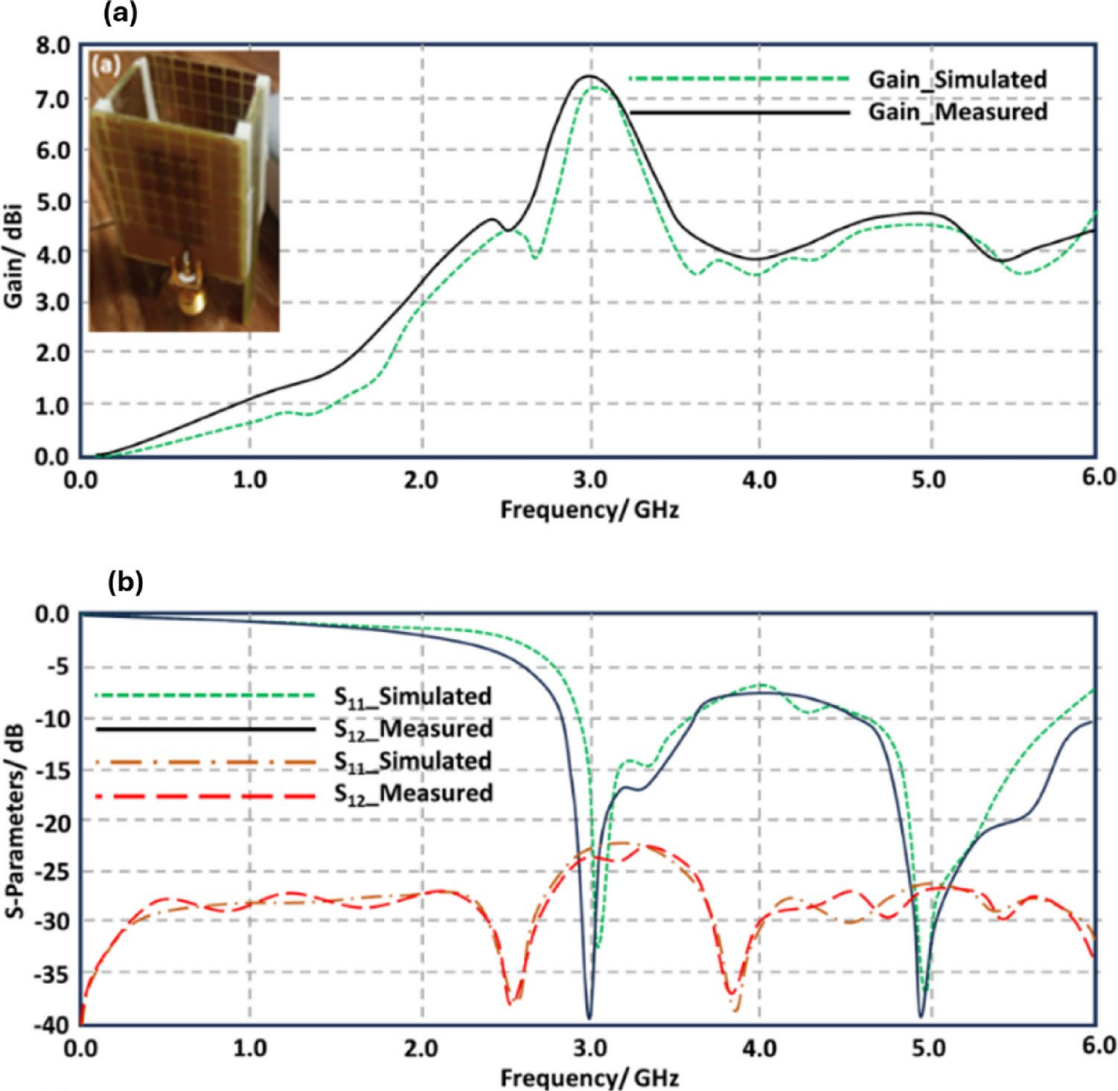
Experimental validation of the proposed antenna: (**a**) comparison between the measured and simulated antenna gain responses, with the inset showing the fabricated prototype, and (**b**) comparison between the measured and simulated reflection coefficient (S_11_) and transmission coefficient (S_21_) responses.

The fabricated two element MIMO antenna configuration is shown in Fig. 10(a). The antenna elements are arranged with CRLH isolation bases placed between them in order to suppress mutual coupling. The MIMO measurements are performed for the 00 switching state, which provides the widest bandwidth and the most stable resonance characteristics.

Figure 10(b) presents the simulated and measured S parameters of the MIMO antenna. The reflection coefficient shows strong impedance matching across the operating bands, while the transmission coefficient remains below − 15 dB throughout the frequency range of interest, confirming effective isolation between the antenna elements. The measured results closely follow the simulated responses, demonstrating the effectiveness of the proposed CRLH isolation structure.

The measured antenna gain is also compared with the simulated results, as shown in Fig. 10(a), and a good agreement is observed. To further evaluate the measurement accuracy, the percentage error between the simulated and measured results is summarized in Table 3. At 3 GHz, the maximum bandwidth error is approximately 3.3%, the isolation error is less than 1%, and the gain error is about 0.7%. These small deviations confirm the reliability of the proposed antenna design and validate the fabrication process.

**Table 3:** The evaluated errors between measured and simulated results around first frequency band.

Parameters @ 3 GHz	Simulated	Measured	Error
S_11_ (dB)	-40	-32	5%
S_12_ (dB)	-23	-22	1%
Bandwidth (GHz)	2.79–3.65	2.86–3.65	3.3%
Gain (dBi)	7.5	7.6	0.7%

### Effect of solar panel integration

**Fig. 11:**
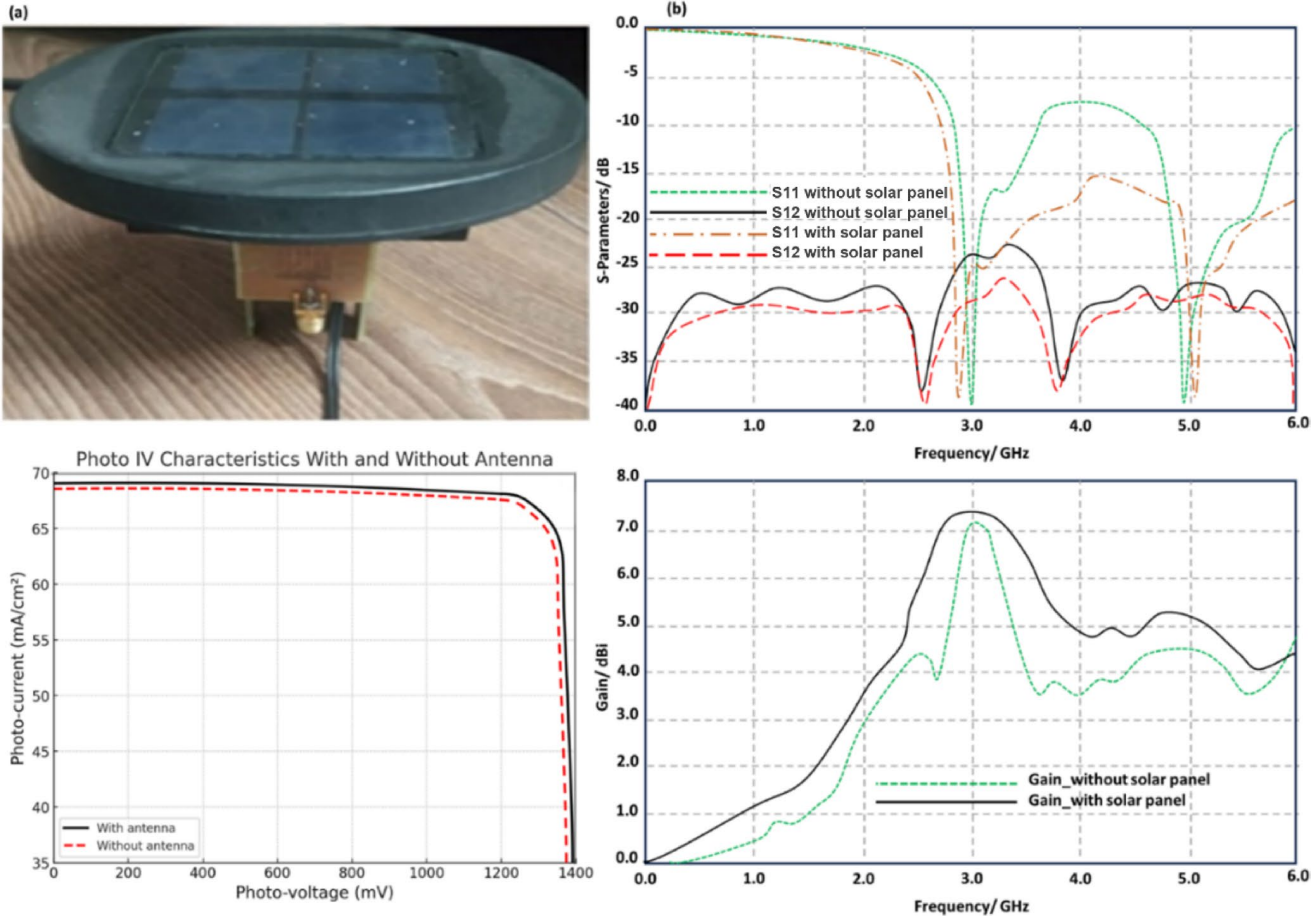
Effect of solar panel integration on antenna performance: (**a**) fabricated antenna mounted beneath a solar panel with measured photo current and photo voltage characteristics, and (**b**) comparison of antenna S-parameters and gain responses with and without solar panel integration.

The effect of integrating the antenna array with a solar panel is investigated to evaluate electromagnetic interaction and energy harvesting compatibility. Figure 11(a) shows the fabricated antenna mounted beneath a planar solar panel along with the measured current voltage characteristics of the photovoltaic module with and without antenna integration.

The measured photo current and photo voltage curves indicate that the presence of the antenna has a negligible impact on the electrical performance of the solar panel. This behavior is attributed to the orthogonal orientation between the antenna radiation direction and the solar panel operating surface, which minimizes electromagnetic interference. The simulated radiation efficiency analysis further shows that the presence of the solar panel causes only a minor variation of approximately 3–5% across the 2.7–6 GHz operating range, indicating that the photovoltaic overlay introduces negligible electromagnetic loss while maintaining stable antenna radiation performance.

Figure 11(b) compares the antenna S parameters and gain responses with and without solar panel integration. The reflection coefficient characteristics remain stable after introducing the solar panel, indicating that impedance matching is preserved. Notably, a measurable gain enhancement is observed when the antenna is integrated with the solar panel. This improvement is attributed to constructive electromagnetic interaction between the antenna and the conductive layers of the solar panel, which effectively act as a reflector and improve radiation directivity.

The results demonstrate that the proposed antenna maintains stable impedance matching, strong isolation, and enhanced gain when integrated with a solar panel. The combined experimental and simulated results confirm that the proposed design is suitable for self powered wireless communication applications without compromising antenna or photovoltaic performance.

### Wireless channel characterization

The proposed MIMO antenna is evaluated in terms of wireless channel performance to examine its behavior in practical communication environments. The analysis includes channel capacity, bit error rate, channel estimation accuracy, and localization performance in order to demonstrate how the antenna characteristics influence system level performance.

#### Channel capacity and bit error rate analysis

**Fig. 12:**
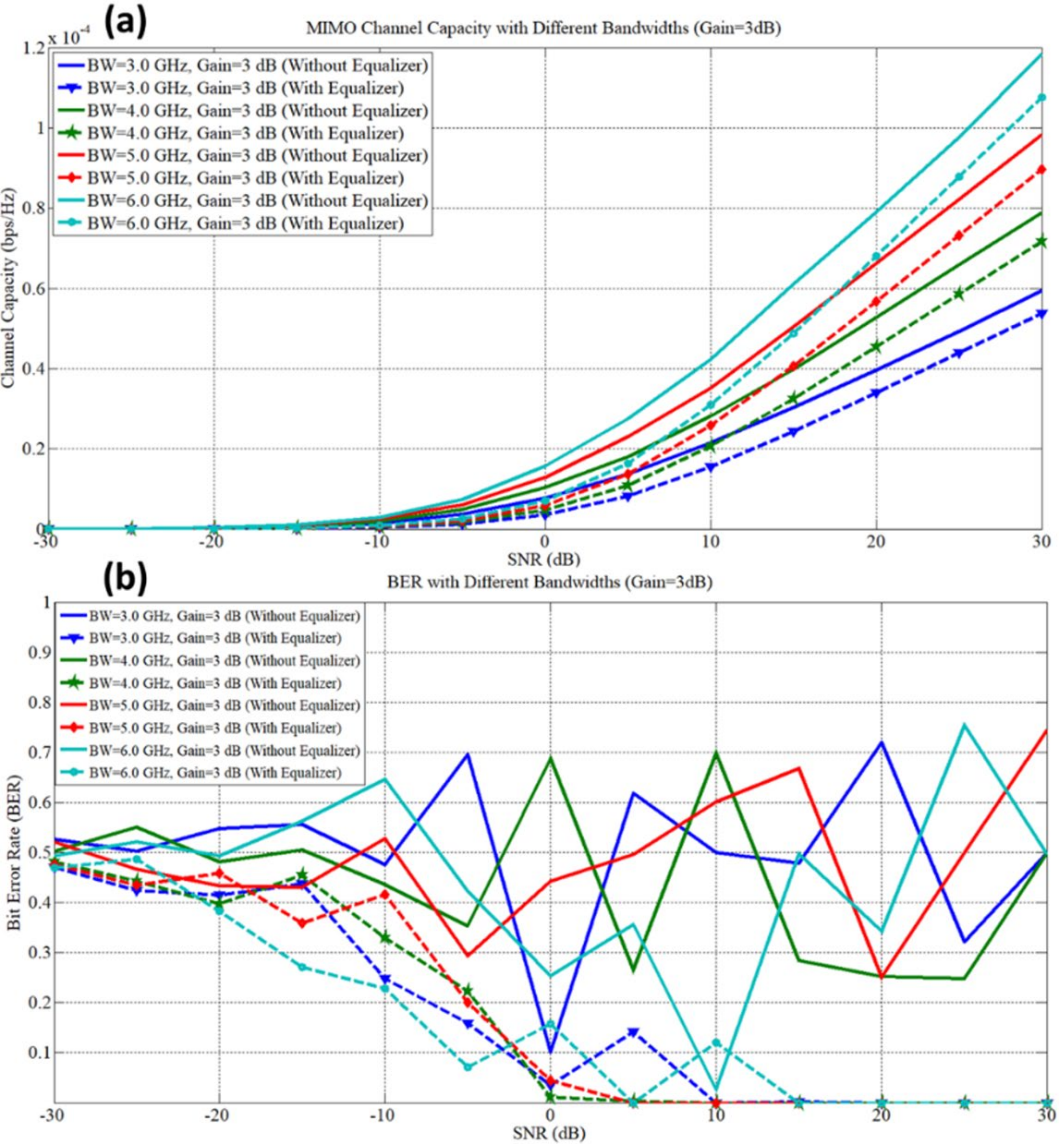
Wireless channel performance of the proposed MIMO system for different bandwidths: (**a**) channel capacity versus signal to noise ratio with and without equalization, and (**b**) bit error rate versus signal to noise ratio for different bandwidth values.

Figure 12 shows the channel performance of the proposed MIMO antenna system for different operating bandwidths while maintaining a constant antenna gain of 3 dB. Figure 12(a) illustrates the channel capacity as a function of signal to noise ratio for bandwidths of 3 GHz, 4 GHz, 5 GHz, and 6 GHz, with and without the use of an equalizer.

The results indicate that channel capacity increases with increasing SNR for all bandwidths, while wider bandwidths achieve higher capacity due to increased available spectrum. The use of an equalizer further improves channel capacity by reducing channel distortion and inter symbol interference. A small reduction in normalized capacity observed in some cases is attributed to the bandwidth overhead required for equalizer training and pilot signaling^24^.

Figure 12(b) presents the corresponding bit error rate performance. Without equalization, the BER remains relatively high due to multipath fading effects. When equalization is applied, the BER decreases significantly as SNR increases, particularly for bandwidths of 4 GHz and above. At high SNR levels, the BER approaches near zero, demonstrating reliable data transmission. These results confirm that the wide bandwidth capability of the proposed antenna contributes to improved communication reliability^23^.

#### Channel tap magnitude and estimation error

**Fig. 13:**
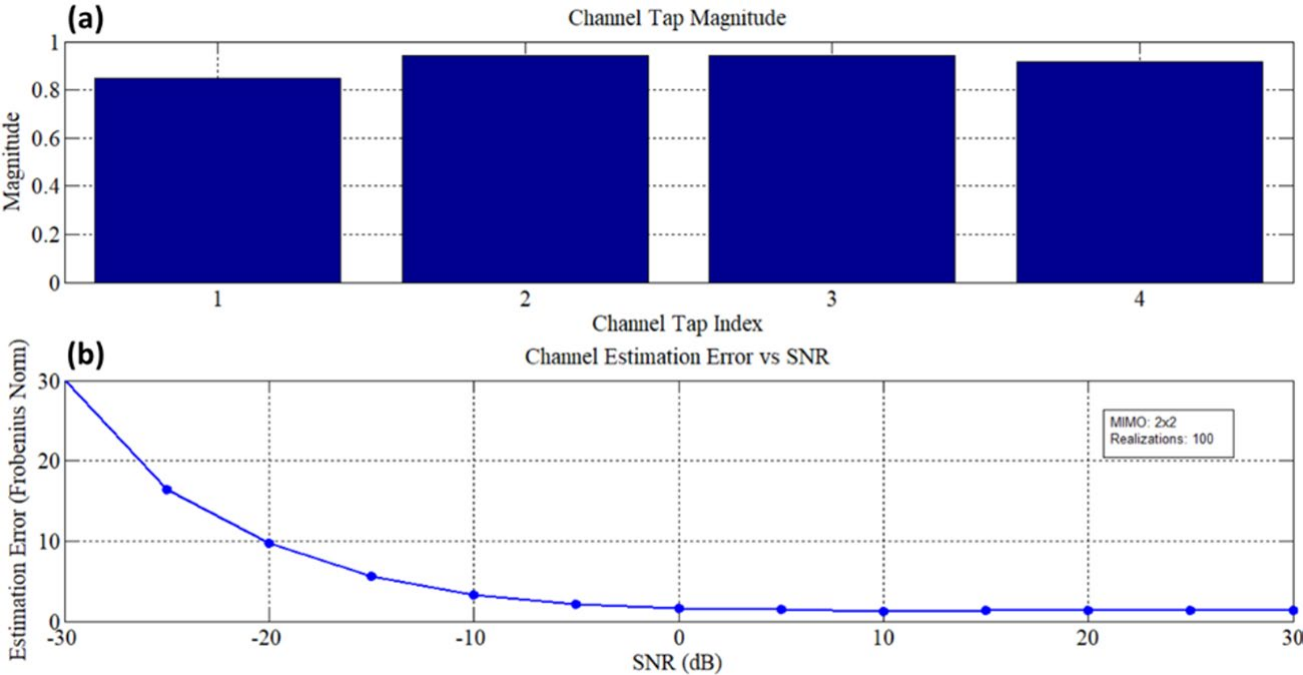
Channel estimation performance for a 2 × 2 MIMO configuration: (**a**) channel tap magnitude distribution, and (**b**) channel estimation error versus signal to noise ratio expressed as Frobenius norm.

Figure 13 presents the channel estimation performance for a 2 × 2 MIMO configuration. Figure 13(a) shows the channel tap magnitudes, where the first tap exhibits the strongest magnitude followed by weaker taps corresponding to multipath components. This behavior is typical in wireless channels where the dominant path carries most of the signal energy^22^.

Figure 13(b) illustrates the channel estimation error expressed as the Frobenius norm versus SNR. At low SNR values, the estimation error is relatively high due to noise effects. As SNR increases, the estimation error decreases rapidly and gradually stabilizes at higher SNR values, indicating improved estimation accuracy^18^.

#### Channel estimation distribution

**Fig. 14:**
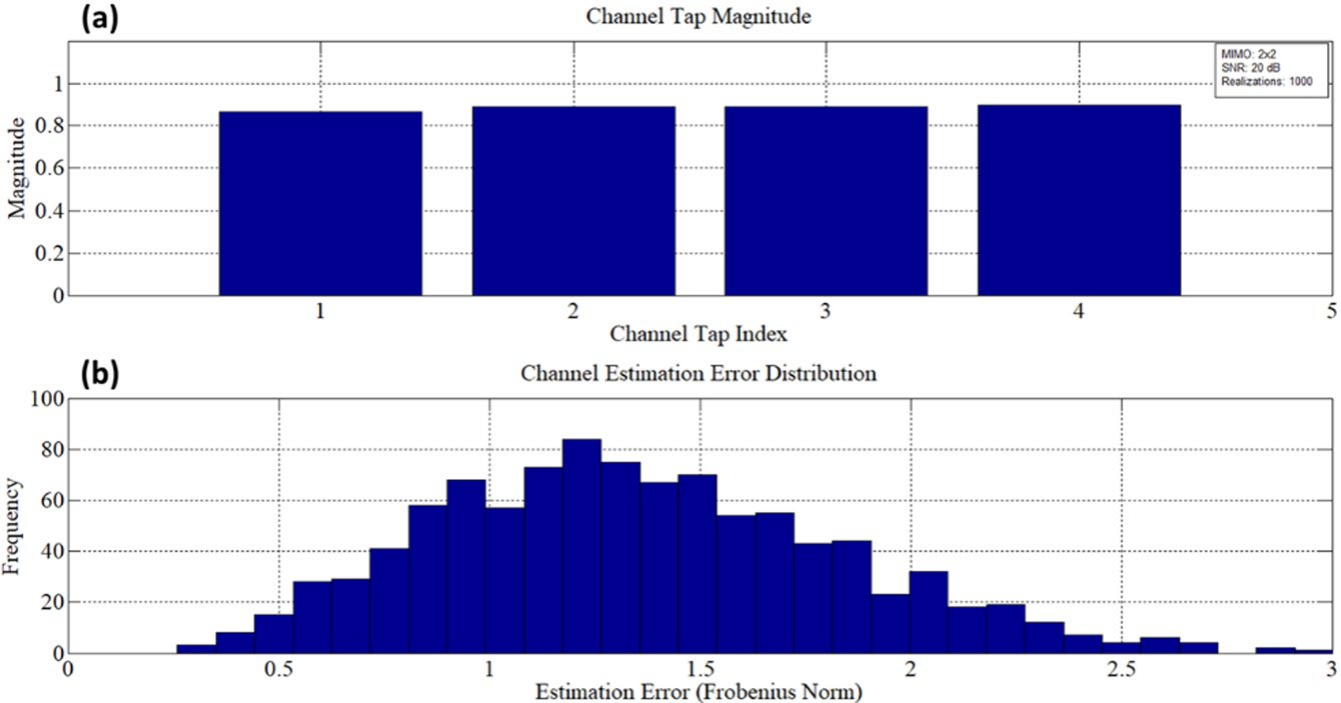
Statistical analysis of channel estimation at 20 dB signal to noise ratio: (**a**) channel tap magnitude for multiple realizations, and (**b**) distribution of channel estimation error.

Figure 14 provides further insight into channel estimation accuracy. Figure 14(a) shows the channel tap magnitudes evaluated at an SNR of 20 dB for multiple realizations, demonstrating consistent multipath behavior. Figure 14(b) presents the distribution of channel estimation errors, where most values are concentrated within a narrow range with a gradually decreasing tail. This distribution indicates that the majority of channel realizations result in low estimation error while larger errors occur less frequently due to deep fading or noise effects^15^.

#### Channel localization performance

**Fig. 15:**
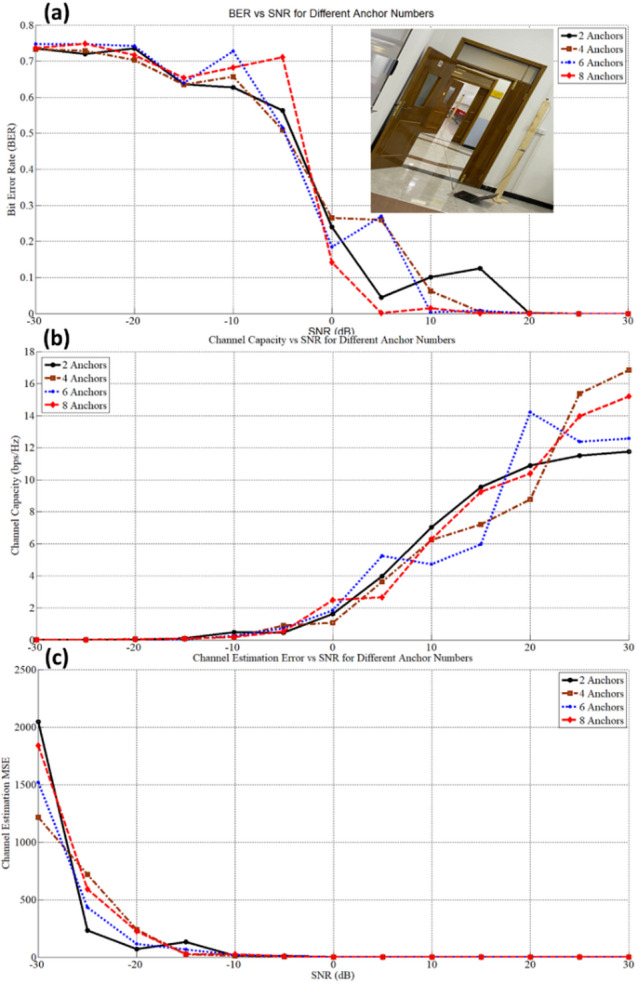
Wireless localization performance of the proposed system for different anchor numbers: (**a**) bit error rate versus signal to noise ratio, (**b**) channel capacity versus signal to noise ratio, and (**c**) channel estimation mean square error versus signal to noise ratio.

The localization performance of the proposed MIMO system is evaluated in Fig. 15 for different numbers of anchor nodes. Figure 15(a) shows the bit error rate as a function of SNR for configurations with 2, 4, 6, and 8 anchors. Increasing the number of anchors improves spatial diversity and leads to a noticeable reduction in BER, particularly at moderate and high SNR values^17^.

Figure 15(b) presents the channel capacity versus SNR for different anchor numbers. The results show that channel capacity increases as the number of anchors increases due to the additional spatial degrees of freedom available for signal transmission^22^.

Figure 15(c) illustrates the channel estimation mean square error versus SNR. The estimation error decreases with increasing SNR, and systems with more anchors achieve lower error levels. At high SNR values, the estimation error approaches near zero, indicating accurate localization performance^14^.

#### Discussion and relation to antenna performance

The wireless channel results demonstrate that the proposed MIMO antenna supports high capacity communication, low error rates, and accurate channel estimation. The wide bandwidth, enhanced gain, and reduced mutual coupling achieved by the antenna contribute directly to the improved channel capacity and BER performance shown in Fig. 12. In addition, stable radiation characteristics and strong isolation enable reliable channel estimation and localization, as illustrated in Figs. 13, 14 and 15.

Compared with the antenna designs summarized in Table 4, the proposed antenna provides a balanced combination of compact size, wide bandwidth, high gain, and strong isolation. These antenna level improvements translate into enhanced system level communication performance while maintaining a compact and energy efficient antenna platform.

**Table 4:** Performance comparison of the proposed MIMO antenna with previously reported designs.

Refs.	Antenna size (λo)^2^	Number of Ports	Frequency BW (GHz)	Maximum Gain (dBi)	Coupling (dB)	ECC	Separation distance
^24^	7.1 × 5.5	8	5.015–5.925	2.1	− 15	0.05	λ/1.9
25	8.2 × 4.1	4	5.15–5.85	1.9	− 14	0.06	λ/2
26	7.4 × 3.3	8	5.15–5.925	1.9	− 10	0.09	λ/2
27	9.1 × 4.5	12	4.8–5.1	2.6	− 12	–	λ/2.1
28	8.1 × 4.3	8	5.147–5.95	2.2	− 10	0.11	λ/2.3
29	8.8 × 8.8	4	3.3–5.8	1.1	− 15	0.10	λ/2.1
30	8.5 × 3.7	2	5.6–5.67	12	− 30	0.06	λ/1.4
This work	3 × 4	4	2.7–6 and beyond	7.3	− 20	0.01	λ/15

## Conclusion

This paper has presented a compact, reconfigurable MIMO antenna system integrated with a solar panel for sub six gigahertz 5G communication applications. The proposed design introduces a novel integration of a Hilbert curve based metamaterial split ring resonator radiating patch, a defected electromagnetic band gap ground plane, and composite right left handed isolation structures, enabling simultaneous enhancement of bandwidth, gain, and mutual coupling suppression within a compact footprint.

A key innovation of this work lies in the combined use of metamaterial radiators and CRLH isolation walls to achieve strong inter element isolation better than minus 15 dB while maintaining wideband operation from 2.7 GHz to 6 GHz and beyond. Frequency reconfigurability is realized through PIN diode switching, allowing dynamic control of resonance behavior and radiation performance without increasing antenna size or complexity. In addition, the antenna array is successfully integrated beneath a solar panel, where experimental results confirm negligible impact on photovoltaic current voltage characteristics and a measurable gain enhancement due to constructive electromagnetic interaction.

Both simulated and measured results show strong agreement, validating the proposed design methodology. The antenna demonstrates stable impedance matching, high gain of up to approximately 7.3 dBi, low envelope correlation, and effective mutual coupling reduction. Wireless channel characterization further confirms that these antenna level improvements translate into enhanced system level performance, including increased channel capacity, reduced bit error rate, and accurate channel estimation and localization.

In comparison with existing designs, the proposed antenna offers a favorable balance between compact size, wide bandwidth, high gain, and strong isolation, while uniquely incorporating solar panel integration and reconfigurability. These features make the proposed antenna a promising candidate for energy efficient and self powered 5G base stations, portable wireless devices, and future sustainable communication infrastructures.

## Data Availability

The data that support the findings of this study are available from the corresponding author upon reasonable request.

## Declarations

All of the figures, materials, and data within the manuscript are original and owned by authors.
